# Thermodynamic Characterization of the Interaction between the Antimicrobial Drug Sulfamethazine and Two Selected Cyclodextrins

**DOI:** 10.3390/molecules24244565

**Published:** 2019-12-13

**Authors:** Hiba Mohamed Ameen, Sándor Kunsági-Máté, Balázs Bognár, Lajos Szente, Miklós Poór, Beáta Lemli

**Affiliations:** 1 Department of General and Physical Chemistry, Faculty of Sciences, University of Pécs, Ifjúság 6, H-7624 Pécs, Hungary; hiba83@gamma.ttk.pte.hu; 2 Institute of Organic and Medicinal Chemistry, Medical School, University of Pécs, Szigeti 12, H-7624 Pécs, Hungary; kunsagi-mate.sandor@gytk.pte.hu (S.K.-M.); balazs.bognar@aok.pte.hu (B.B.); 3 János Szentágothai Research Center, University of Pécs, Ifjúság 20, H-7624 Pécs, Hungary; poor.miklos@pte.hu; 4 CycloLab Cyclodextrin Research & Development Laboratory, Ltd., Illatos 7, H-1097 Budapest, Hungary; szente@cyclolab.hu; 5 Department of Pharmacology, Faculty of Pharmacy, University of Pécs, Szigeti 12, H-7624 Pécs, Hungary

**Keywords:** cyclodextrin, sulfamethazine, zwitterion, host-guest complex, thermodynamics

## Abstract

Sulfamethazine is a representative member of the sulfonamide antibiotic drugs; it is still used in human and veterinary therapy. The protonation state of this drug affects its aqueous solubility, which can be controlled by its inclusion complexes with native or chemically-modified cyclodextrins. In this work, the temperature-dependent (298–313 K) interaction of sulfamethazine with native and randomly methylated β-cyclodextrins have been investigated at acidic and neutral pH. Surprisingly, the interaction between the neutral and anionic forms of the guest molecule and cyclodextrins with electron rich cavity are thermodynamically more favorable compared to the cationic guest. This property probably due to the enhanced formation of zwitterionic form of sulfamethazine in the hydrophobic cavities of cyclodextrins. Spectroscopic measurements and molecular modeling studies indicated the possible driving forces (hydrophobic interaction, hydrogen bonding, and electrostatic interaction) of the complex formation, and highlighted the importance of the reorganization of the solvent molecules during the entering of the guest molecule into the host’s cavity.

## 1. Introduction

Formation of host-guest type inclusion complexes typically occurs when the host molecule uses its cavity to encapsulate a guest through noncovalent interactions. According to the significant practical utility of macrocyclic molecules, such as calixarenes [1,2], cavitands [3,4], and cyclodextrins (CDs) [5,6] in host-guest complex formations, chemists, biologists, and material scientists got interested the physical properties, chemical nature, and related biological activity of these molecules. However, utilization of these noncovalent interactions (hydrogen-bonding, π-stacking, electrostatic interaction, van der Waals force, and hydrophobic/hydrophilic attraction) are still a great challenge [7,8,9,10]. CDs, a fascinating class of macrocycles, are composed of six, seven, or eight glucose units, called α-, β-, and γ-CDs, respectively. CDs are used as host components for the construction of various interesting supramolecular structures [11,12]. Sulfonamide antibiotics are widely used in both human medicine and livestock production to treat some bacterial infections of the urinary tract, ears, lungs, skin, and soft tissues [13,14]. Furthermore, sulfonamides can appear as contaminants in various foods, which may cause adverse health effects [15,16,17]. The host-guest type complex formation of these antibiotics with CDs is an extensively studied field [18,19,20,21,22,23,24]. Zoppi et al. focus on the increased water solubility of sulfonamide drugs in the presence of native and methylated β-CD [23,24]. In the case of sulfamethazine (SMT), their nuclear magnetic resonance (NMR) and molecular modeling results demonstrate that SMT included the substituted pyrimidine ring into the β-CD cavity. Contradictory, NMR and quantum chemical results of Bani-Yaseen and Mo’ala revealed that complex formation is favorable with inclusion of the aniline moiety through the β-CD cavity [18]. 

Several studies have been performed to get an insight into the factors which affect the thermodynamic and kinetic stability or selectivity of host-guest complexes [25,26], because the deeper understanding of these interactions has high importance. The pH-responsive host-guest encapsulation is also a highly studied field in material sciences [27] and in pharmacology [28,29]. Therefore, besides the complex stability and stoichiometry of SMT – β-CD complex and along the contradictory description of the related structures [18,23,24], the investigation of the pH dependence interaction of SMT with CDs is also reasonable. 

**Figure 1:**
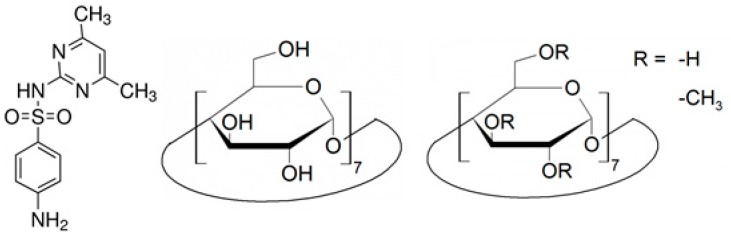
Chemical structures of sulfamethazine (SMT), native β-cyclodextrin (BCD), and randomly methylated β-cyclodextrin (RAMEB).

In our recent study [30], we demonstrated the importance of pH-dependent dipole moment of SMT molecule, which phenomenon can affect the complex geometry formed with β-CD (BCD) and randomly methylated β-CD (RAMEB) (Figure 1). Now we focus on the thermodynamic properties of the formation of inclusion complexes at different pH values. Our aim is to analyze the weak interactions between the pH dependent ionic and neutral forms of SMT and native or methylated CDs at molecular level to clarify the previous contradictory results. In this way, the involvement of weak molecular interactions (electrostatic forces and hydrogen bonds) have been tested by the temperature-dependent measurements and molecular modeling studies. 

## 2. Results and Discussion

### 2.1. Temperature Dependence of the Association Constants of Sulfamethazine-CD Complexes at Different pH

**Figure 2:**
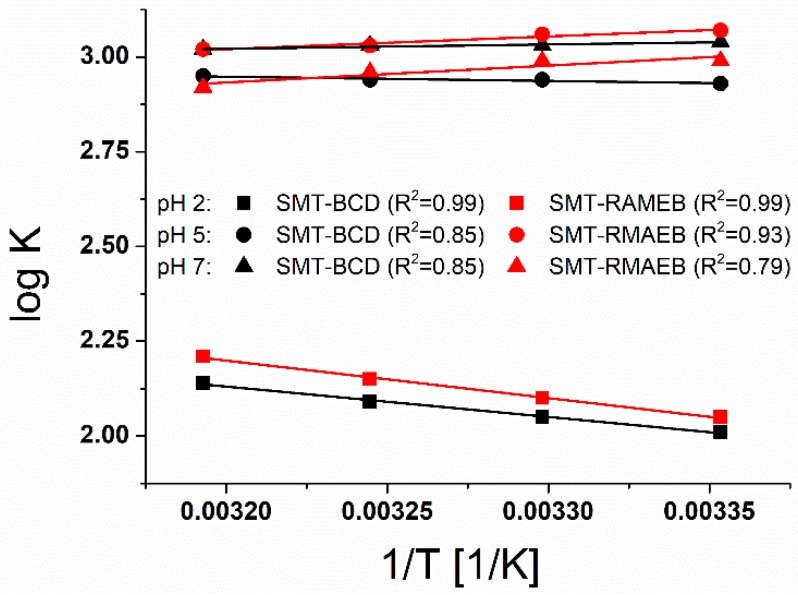
The van’t Hoff plots of SMT-BCD and SMT-RAMEB complex formations at different pH values.

Figure 2 shows the van’t Hoff plot of SMT-CD complexes, based on association constants determined at different temperatures. In accordance with our earlier findings [30], significant difference between the association constants at elevation pH (pH = 5 and pH = 7) and at strong acidic environment (pH = 2) has been found. The slight dependence of complex stabilities on the temperature reflects low enthalpy changes, i.e., weak interactions between the molecules. At pH 7, where the nonionic and anionic guest molecules are dominant, higher stability is associated to the complexes at decreased temperatures. In contrast, in the presence of considerable amount of cationic guest at pH 2, the complex stability increases with the elevation of the temperature. Although, only one form of the guest molecule (nonionic SMT) is available at pH 5, the substitution of the β-CD affects the change of the association constants with the temperature. The association constants of CD complexes generally decrease with the elevation of the temperature [19,31]. However, one of our earlier work showed an opposite example [32]. The thermodynamic parameters have been also determined to analyze further the related processes.

Thermodynamic parameters (Table 1) were calculated from the slopes and the intercepts of the lines fitted to the experimental data based on the van’t Hoff plot (Equation (1), see Figure 2). The negative ΔG values yield spontaneous complex formation between SMT and CDs. Results showed exothermic association at high pH (pH = 7), while an endothermic molecular association was obtained at low pH (pH = 2). At pH 5, the endothermic character of the complex formation was just changed to exothermic as a result of the methyl substitution of BCD. In each interaction, an entropy gain was observed; however, the entropy increase during the complex formation correlates with the enthalpy change. The entropy increase during the association reaction was probably due to the process when SMT enters the CD cavity (it releases its solvation shell). Furthermore, higher entropy gain associated with positive or less negative enthalpy, which property reflects to the removal of more or less water molecules from the solvation shell regarding the molecules interacted during formation of complexes. Decreased ΔS at higher pH values suggest the release of less water molecules from the solvation shell of SMT molecules, because the stabilization is also supported by the attractive coulomb forces between the negatively charged SMT and the dipole moments of the solvent molecules. The correlation between the enthalpy and the entropy changes can be described by the changes of the solvation shell of guests, since the removal of less water molecules from the solvation shell costs less energy. This description agrees with the enthalpy-entropy compensation and highlights that the exothermicity of molecular association usually restricts the movement of the constituents, thereby causing growing entropy loss.

### 2.2. Modeling Studies

**Figure 3:**
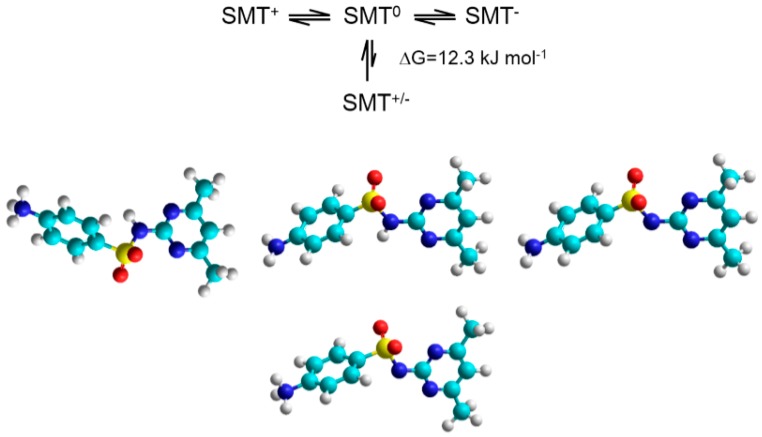
The energetically most favorable deprotonation routes of SMT (cationic: left, nonionic: middle, anionic: right, zwitterionic: bottom) determined by MINDO/3 approximation using the TIP3P solvation model for the buffer [34]. Gibbs free energy between the nonionic and zwitterionic forms suggest presence preferably of nonionic form in the solution.

**Figure 4:**
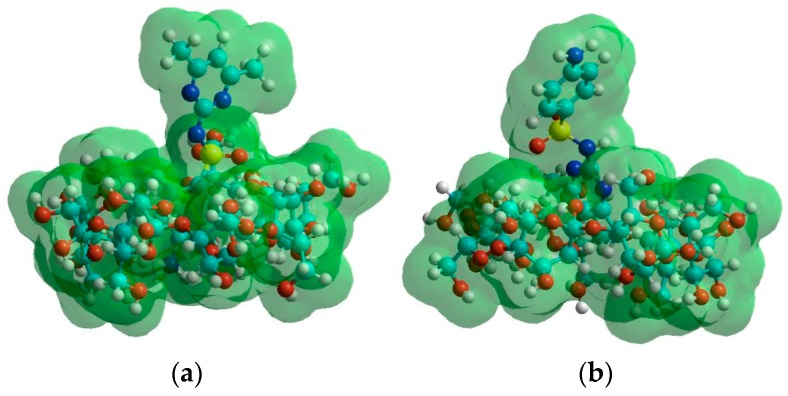
Equilibrium conformation of SMT-BCD complexes. (**a**) SMT molecules with their aromatic amine moiety and (**b**) with their methyl groups enter into the cavities of hosts.

To get a deeper insight into the complex formation processes, molecular modeling studies were performed at semi-empirical level. During these calculations, the energetically favorable deprotonation route of SMT molecule was determined first in aqueous solutions considering the presence of other ions as described in the Materials and Methods section. Sulfamethazine exists as cationic (SMT^+^), anionic (SMT^−^), nonionic (SMT^0^) and zwitterionic (SMT^+/−^) forms in aqueous solutions. Figure 3 shows that the aromatic amine moiety, which is protonated at low pH loses first the proton while the second deprotonation occurs at the sulfonamide nitrogen. The associated experimental pKa_1_ and pKa_2_ values at room temperature were 2.07 and 7.49, respectively. We should mention here that the Gibbs free energy difference between the nonionic and zwitterionic forms of SMT was found to be 12.3 kJ mol^−1^ in this environment. This result suggests presence of SMT in nonionic rather than zwitterionic form in the solutions, however, it is known that zwitterionic form can stabilized e.g., in adsorbed state [33]. Then the interactions of these three forms of SMT (cationic, nonionic and anionic) were examined with BCD and RAMEB host molecules in the aqueous buffer. Due to the huge computation time of the large systems, in the case of RAMEB the electron releasing property of the methyl groups was considered as negatively charged specie of the native BCD molecules. Thus, the repulsive Coulomb interaction between the negatively charged RAMEB cavity (simulated by −1 BCD) and the deprotonated SMT species will reduce the secondary interactions between the host and guest molecules. In contrast, the charged SMT species showed stronger interactions with the negatively charged cavity of RAMEB. Furthermore, the host molecules formed even more stable complexes with the anionic form of the guest. From the point of view the enthalpy (Table 2), the following process is responsible for these unexpected results: at low pH the cationic SMT molecule enters into the host cavity with its aromatic amine moiety. However, at higher pH, SMT molecule enters with its methyl substituents. In the former cases, hydrogen bridges between the (guest amine) N-H ··· O (host hydroxyl), while in the latter cases, the hydrogen bridges between the (guest methyl) C-H ··· O (host hydroxyl) are moderate the weak interactions between the host and guest (Figure 4). Noted here, that this pH dependent orientation of guest molecule in the complexes support the earlier described structures based on the inclusion of the aniline moiety [18] as well as the pyrimidine ring [24] through the CD cavity. 

**Figure 5:**
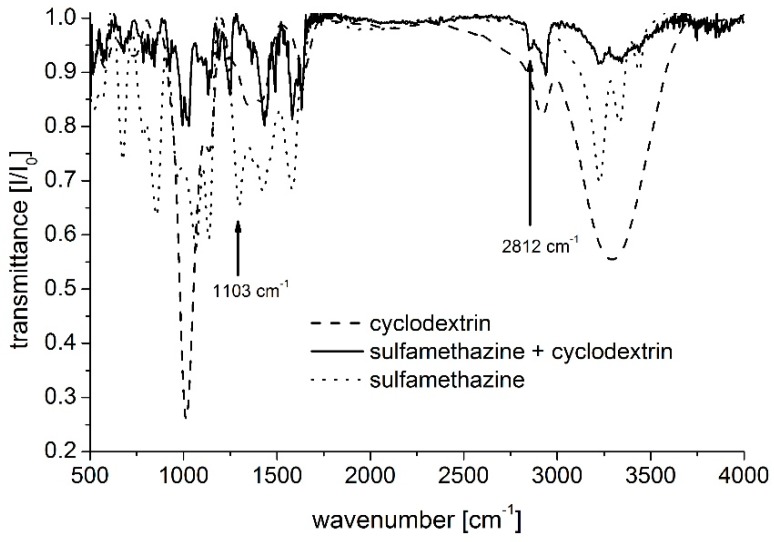
Infrared spectra of SMT – RAMEB complexes.

Furthermore, the inclusion of SMT by its aromatic amine moiety in case of RAMEB host enhances formation of zwitterionic form of SMT in the cavity. This is due to the tautomerization of the proton from the sulfonamide to the aromatic amine moiety enhanced by the Coulomb interaction of the proton with the negatively charged cavity of RAMEB. With the aim to justify this conception of the SMT’s zwitterion formation in the RMAEB’s cavity, simultaneous analysis of complexation behavior has been done using infrared (IR) spectroscopy. In general, our results are in agreement with the IR analyses of SMT-BCD complexes prepared by a freeze-drying method [23], the characteristic bands of SMT shifted and are more or less intense in the presence of CD. Moreover, IR spectra of the SMT-RAMEB complexes and the species interacted support our idea described above (Figure 5): significant changes of two characteristic vibrations of SMT molecules were observed upon complexation by the RAMEB host as follows. Quantum chemical analysis revealed that belting vibration of SNH bond angle at sulfonamide moiety (1103 cm^−1^) disappeared while the bond stretching associated to the aromatic NH_3_ is appeared at 2812 cm^−1^ in the experimental IR spectra of the complexes. These changes in the experimental IR spectra indicate the stabilization of the zwitterionic form of SMT in the RAMEB cavity. This phenomenon has not been observed in the case of the BCD host.

In all eight situations, the interactions show an increased entropy term (Table 2). This property is associated with two facts: the solvent water molecules leave the host’s cavity prior to the complex formation and the guest molecules (at least partly) lose their hydration shell. Both processes increase the entropy. In particular, the entropy gain decreases by the second deprotonation step. This is probably due to the increase in the stability of the hydration shell regarding the anionic SMT molecules.

Considering that the formation of hydrogen bridges between the host and guest always assumes dehydration of the appropriate part of the host and guest molecules, the energy cost of dehydration compensated by the entropy gain associated to the increased freedom of the water molecules after the dehydration. This assumption is supported by the good agreement between the measured and calculated thermodynamic parameters.

### 2.3. Driving Forces of the SMT-CD Complex Formations

Taking into account the binding conformations suggested by theoretical modeling, we can discuss in detail the thermodynamic parameters of complex formation between CDs and SMT. However, it should be noted here, that thermodynamic parameters derived from temperature- dependent spectroscopic measurements assume that these parameters are constants within the temperature range of investigation. Furthermore, these data reflect not only for the temperature-dependent change of the association constants, but also for the way how the association constants have been determined. Spectroscopic identification of association constant based on changes of the environment around the guest when the molecule enters from the polar aqueous media the hydrophobic cavity of the CD. Therefore, the related enthalpy changes and entropy changes describe the complex formation without solvent interaction in the bulk phase. Isothermal titration calorimetry is the accurate technique to solve this problem and to measure directly thermodynamic properties of host-guest complex formation. However, in this work, the thermodynamic parameters were determined based on fluorescence spectroscopic measurements, using the van’t Hoff equation. Relevant experiments showed [19] that the results of calorimetric studies are similar to the spectroscopic findings regarding host-guest type CD complexes, and the data of thermodynamic parameters only slightly differs between the two methods. This property supports our conclusions made on the spectroscopic data.

The possible driving forces which stabilize the host-guest complexes of CDs are electrostatic interaction, van der Waals interaction, hydrophobic interaction, hydrogen bonding, relief of conformational strain, charge transfer interaction, and release of water molecules from the hydrophobic cavity of the host to the bulk phase [35]. The values of thermodynamic parameters consist the contribution of the species’ desolvation and the different kind of noncovalent interactions listed above. In general, the combination of both negative or positive enthalpy and entropy changes indicate that van der Waals forces and hydrogen bonding or hydrophobic interaction take places in complex formation, respectively. While higher negative values of ΔH combined with positive ΔS have found for the electrostatic driving forces combined with hydrogen bonds of ionized groups [36]. However, the given values can be strongly affected by intensive dehydration and solvent reorganization. In the discussion of the present experimental data (Table 1) we focus on two tendencies observed in the thermodynamic parameters: both the enthalpy and entropy changes associated to the complex formation decrease while the charge of the guest SMT molecules varies from +1, 0 to −1. On this base, considering the attractive forces between the anionic cavity of the host and the cationic guest at pH = 2, highly negative enthalpy changes should be observed in vacuo. However, the desolvation of the guest costs more energy than it is causes during the association of SMT with BCD, therefore a positive enthalpy change can be observed. The ordered structure of solvent molecules in the solvation shell is destroyed after the complex is formed and the free solvent molecules gain the entropy. Results related to the complexation of the neutral form at pH = 5 suggest preference of the latest effect: weaker stability of the solvation shell assumes much lower energy costs for its destroying, therefore the enthalpy change lowered instead the weaker contribution of the attractive coulomb forces. As parallel effect on the entropy, weaker stability of solvent molecules in the solvation shell of the guest assumes higher entropy content of the solvent molecules prior complex formation which property causes lower entropy gain during the interaction with the CD hosts. The complex formation, however, is also affected by the formation of zwitterion of the guest at pH = 5 and this property enhances the decrease of the enthalpy when the positively charged NH_3_ group of SMT interact the more negatively charged cavity of the RAMEB while the negative sulfonamide nitrogen of SMT interact with the positively charged methyl groups of the host. These three processes (coulomb interaction, desolvation of the guest prior formation of the complex and the formation of zwitterionic derivative of the SMT) will then compete. At pH = 7 comparable amount of neutral and anionic form of SMT are presented in aqueous solution. The further decrease of both the enthalpy and entropy changes associated to the complex formation highlighted the complex stabilization effect of the deprotonated sulfonamide nitrogen. Presence of competition of the processes above was then confirmed by the analysis of enthalpy – entropy compensation.

**Figure 6:**
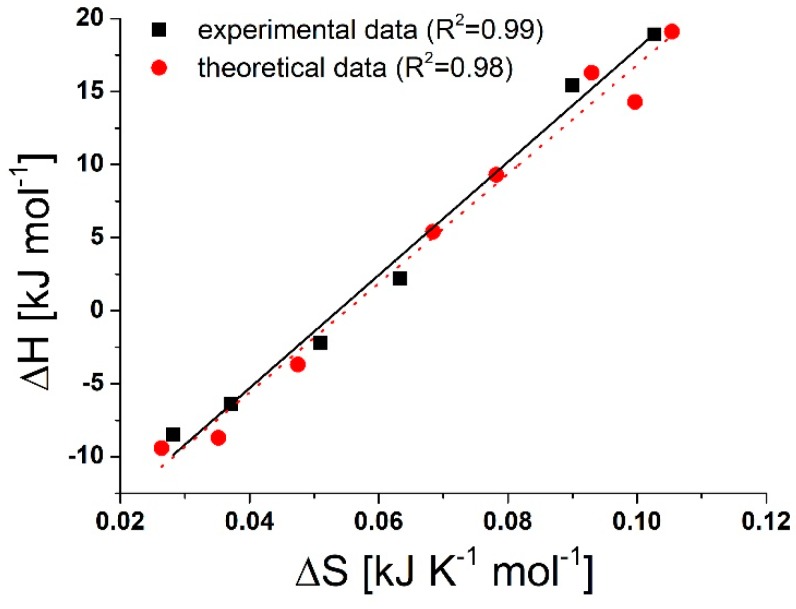
Enthalpy-entropy compensation plot of SMT-BCD and SMT-RAMEB complexes.

The enthalpy-entropy compensation is still a widely observed and unresolved phenomenon in chemical thermodynamics [37,38,39]. The linear correlation when the experimentally found ΔH and ΔS values are plotted against each other is believed to play an important role in the formation of weak interactions. However, for similar systems, the Gibbs free energy remains the same. Figure 6 shows the ΔH vs. ΔS plots for SMT-CD complexes analyzed in the present work. Both experimentally and theoretically determined data are presented. Although the processes have been investigated in a small temperature range (298–313 K), the compensation temperature determined from the slope of the good straight line (387 K and 374 K experimental and theoretical data, respectively) are far to the average temperature. This small difference could arise from the indirect determination of the thermodynamic parameters based on spectroscopic measurements. The difference between the ΔG values (~6.1 kJ mol^−1^ in the present systems) brings the experimental and compensation temperature farther [38]. In biological supramolecular systems, also in CD chemistry, the studies of enthalpy-entropy compensation have been started early and it has been widely investigated. Twenty years ago, a review comprises more than 1000 thermodynamic data of the inclusion complexes of native and chemically-modified CDs [40]. Based on the analyzes of the enthalpy-entropy compensation plot of native and modified CDs or the α-, β- and γ-CDs, it was found that the linearity and the slope of the straight line could be affected by the difference between the conformational change of the native and modified CDs, by the desolvation of both host and guest molecules, and by the ring size and flexibility. However, in recent studies [35,37], the compensation effect is mainly interpreted based on the changes in the level of hydration and reorganization of the solvent molecules. The considerable effect of the solvent is not surprising, since solvation known to affect the electronic structure of molecules, so affects the interactions between electrons of different atomic or molecular orbitals. Therefore, it affects also the molecular interactions, especially when they are weak [41,42]. In the present case, if the anionic guest molecule keeps the part of its solvation shell, then the higher ordered structure of the complexes (included by its solvation shell) explain the deprotonation enhanced entropy gain decreases. Because there is no significant difference between the cavity size and flexibility of BCD and RAMEB and the enhanced electron rich character of the methylated CD should result in opposite effect than we have found, the small entropy term differences can be explained by poor solubility of native BCD (owing to the highly ordered water molecules in its solvation shell) [43]. When the guest molecule enters into the CD cavity, the interaction (at least partly) destroys the solvation shell of the host and weakens the CD-solvent interaction. Similarly to our earlier findings [32], when the solvent molecules leave the host’s cavity, reorganization of the more ordered BCD-water structure results in a higher entropy change vs. the less ordered RAMEB-water system. 

## 3. Materials and Methods 

### 3.1. Reagents

Sulfamethazine (SMT) was purchased from Alfa Aesar (Kandel, Germany). Stock solutions of SMT (5000 µM) were prepared in methanol (spectroscopic grade, Reanal, Budapest, Hungary). Diluted solutions of SMT were prepared by evaporating the methanol under relatively low pressure, then SMT was dissolved in appropriate volumes of the phosphate buffer of interest. CDs, including β-cyclodextrin (BCD) and randomly methylated β-cyclodextrin (RAMEB) were obtained from CycloLab Cyclodextrin Research and Development Laboratory, Ltd. (Budapest, Hungary). All the other analytical grade chemicals were purchased from VWR International Ltd. (Debrecen, Hungary). Phosphate buffer solutions have been prepared by mixing (0.1 M) H_3_PO_4_ and (0.1M) Na_2_HPO_4_, (0.01 M) H_3_PO_4_ and (0.01 M) Na_2_HPO_4_ or (0.01 M) KH_2_PO_4_ and (0.01 M) Na_2_HPO_4_ stock solutions until the requested pH 2, 5 or 7 were reached, respectively. Ultrapure water (conductivity < 0.1 μS/cm,) were prepared by an Adrona (Riga, Latvia) water purification system. 

### 3.2. Fluorescence Spectroscopic Studies

Highly sensitive Fluorolog tau3 spectrofluorometer (Jobin-Yvon/SPEX, Longjumeau, France) was used to investigate the fluorescence spectra of the different solutions. For data collection, the photon counting method with 0.1 s integration time was used. Excitation and emission bandwidths were set to 4 nm. A 10 mm thickness of the fluorescent probes with right-angle detection was applied. Temperature-dependent steady-state fluorescence spectroscopic measurements were carried out at different temperatures: 298.2 K, 303.2 K, 308.2 K, and 313.2 K. The fluorescence emission spectra of SMT (30 μM) was recorded in the absence and presence of increasing concentration of BCD or RAMEB (0–3 mM) in different phosphate buffers, using 280 nm excitation wavelength. Similarly, to our previous studies [30,44,45], overall and stepwise association constants of the complex formation were calculated by non-linear fitting, based on the fluorescence emission data obtained, employing the HyperQuad2006 program package [46].

To determine the thermodynamic parameters, temperature dependence of the complex stabilities was examined. According to the van’t Hoff Equation (1) the temperature-dependence of the association constants offers possibility to determine the thermodynamic parameters related to the formation of the SMT-BCD and SMT-RAMEB complexes:

(1) $$lnK=-\frac{\Delta G}{RT}=-\frac{\Delta H}{RT}+\frac{\Delta S}{R},$$

where the Δ*H* and Δ*S* stand for the enthalpy and entropy changes of the complex formation, while Δ*G* is the Gibbs free energy change. *R* stands for the gas constant, while *T* is the temperature in Kelvin.

### 3.3. Infrared Spectroscopy

Fourier transform infrared spectra of SMT, BCD, RAMEB and SMT-BCD and SMT-RAMEB complexes were recorded on Platinum Alpha T FT-IR Spectrometer (Bruker, Ettlingen, Germany). Droplets of samples is used for these measurements. Average of ten scans with 5 cm^−1^ resolution is applied.

### 3.4. Modeling Studies

The thermodynamic parameters of the SMT-BCD or SMT-RAMEB complexes were determined at 298 K as follows: The enthalpy change considered as the energy change calculated by subtracting the total energies of the reactants from the total energies of the products. Similarly, the entropy changes were calculated by subtracting the entropy terms of the reactants from the entropy terms of the products. The entropy terms of the species interacted were calculated applying Boltzmann statistics. The higher contribution to the entropy comes from the vibrational motions. Therefore, after calculating the vibrational frequencies using the harmonic approximation, the entropy was then determined as the following equation implemented in the HyperChem code:

(2) $$S_{vib}=R\sum_{i}\{\frac{h\nu_{i}/kT}{e^{\left(h\nu_{i}/kT\right)}-1}-\ln[1-e^{\left(-h\nu_{i}/kT\right)}]\}$$

where the *ν_i_* is the frequency of vibration and *T* is the temperature (298.16 K). 

The total energies of the species interacted have been calculated at semi-empirical MINDO/3 level using HyperChem 8 code. After the geometry optimization at MINDO/3 level the vibrational-rotational analysis was performed in harmonic approximation using AM1 approximation. Neutral aqueous environment was considered by the TIP3P solvation model implemented in HyperChem code [47]. Considering that in the present studies ionic species are interacted, the ionic strength of the buffer were considered by the additional PO_4_^3−^, K^+^, Na^+^ and H_3_O^+^ ions as described in an earlier study [34]. Accordingly, the final cube for representing solvents has 30 Å × 30 Å × 30 Å sizes and contained water, PO_4_^3−^, HPO_4_^2−^, K^+^, Na^+^ and 9 H_3_O^+^ according to the composition of the buffer solution while the pH varied from 7, 5 and 2. After 10 ps MD simulation to equilibrate the system at room-temperature at MM+ level, the calculations for the complexes and the separated species interacted were performed at MINDO/3 level. To reduce the huge computational time, the random-methylated CD derivative (RAMEB, which have electron-rich cavity) was considered as negatively charged species of the native BCD [48,49]. 

## 4. Conclusions

The complex formation between different sulfonamides and cyclodextrins still has attract much attention. Previous studies described the ability of cyclodextrin to increase the solubility of these drugs in water [23,24]. Subsequently, efforts were made to study the structure of the complexes by experimental and molecular modelling techniques [18,23,24]. Earlier studies focus on the buffer free solution, suspension or freeze-dried solid state complexes. According to our present knowledge our work is the first study to describe the sulfamethazine–β-cyclodextrin and sulfamethazine–randomly methylated β-cyclodextrin complexes in aqueous solution at different pH and temperature values using combined experimental and theoretical techniques. Both spectroscopic measurements and molecular modeling studies highlight the importance of the reorganization of the solvent molecules during the guest molecule enters the host’s cavity. Results highlight formation of zwitterionic sulfamethazine molecule in the cyclodextrin cavity which affect significantly the stability of SMT-CD complexes. The pH-affected structures of the complexes investigated explain the previous contradictory findings. The presented results might relevant for the preparation of orally administreted products of sulfamethazine-cyclodextrin complexes.

## Figures and Tables

**Table 1:** Thermodynamic parameters associated to the formation of SMT-CD complexes. Data are determinate based on temperature-dependent fluorescence spectroscopic measurements. (ΔH [kJ mol^−1^], ΔS [J K^−1^ mol^−1^] ΔG298K [kJ mol^−1^]).

Host Species	pH								
	2			5			7		
	ΔH	ΔS	ΔG_298K_	ΔH	ΔS	ΔG_298K_	ΔH	ΔS	ΔG_298K_
BCD	15.4 ± 0.8	90.0 ± 2.5	−11.4 ± 1.5	2.2 ± 0.5	63.3 ± 1.7	−16.7 ± 1.0	−2.2 ± 0.5	51.0 ± 1.7	−17.3 ± 1.0
RAMEB	18.9 ± 0.8	102.7 ± 2.6	−11.7 ± 1.6	−6.4 ± 1.0	37.2 ± 3.3	−17.5 ± 2.0	−8.5 ± 1.2	28.8 ± 3.8	−17.1 ± 2.3

**Table 2:** Thermodynamic parameters associated to the formation of SMT-CD complexes. Semiempirical MINDO/3 method with TIP3P solvation model is applied. (ΔH [kJ mol^−1^], ΔS [J K^−1^ mol^−1^]).

Host Specie	Host Simulated as	Guest’s Charges							
		+1 (Cationic)		0 (Nonionic)		0 (Zwitterionic)		−1 (Anionic)	
		ΔH	ΔS	ΔH	ΔS	ΔH	ΔS	ΔH	ΔS
BCD	0 BCD	16.3	93.0	9.3	78.2	5.4	68.4	−3.7	47.5
RAMEB	−1 BCD	19.1	105.4	14.3	99.7	−8.7	35.2	−9.4	26.4
